# Calciphylaxis in End-Stage Renal Disease: A Case of Nonhealing Ulcer and Diagnostic Challenges

**DOI:** 10.7759/cureus.84300

**Published:** 2025-05-17

**Authors:** Dina Sulit, Kashmira Jeeva, Mahmoud Abouibrahim, Mansoor Zafar, Zainab Alshiekh Ali, Stefano Berliti

**Affiliations:** 1 Dermatology, James Paget University Hospitals NHS Foundation Trust, Great Yarmouth, GBR; 2 Internal Medicine, East Sussex Healthcare NHS Trust, Conquest Hospital, Hastings, GBR; 3 Endocrinology and Diabetes, James Paget University Hospitals NHS Foundation Trust, Great Yarmouth, GBR; 4 Gastroenterology, East Surrey Hospital, Surrey and Sussex Healthcare NHS Trust, London, GBR; 5 Histopathology, Eastbourne District General Hospital, East Sussex Healthcare NHS Trust, Eastbourne, GBR; 6 Acute Medicine, East Sussex Healthcare NHS Trust, Conquest Hospital, Hastings, GBR

**Keywords:** calciphylaxis, cellulitis, end-stage renal disease (esrd), warfarin-induced skin necrosis, warfarin therapy

## Abstract

Calciphylaxis is a rare, life-threatening condition characterized by calcification, fibrosis, and thrombosis of small- to medium-sized dermal vessels, leading to painful skin lesions and necrosis, primarily in patients with end-stage renal disease (ESRD). We present the case of a 76-year-old male patient with ESRD on hemodialysis who developed a nonhealing, necrotic ulcer over the posterior aspect of his right leg. Clinical features, laboratory findings, and histopathological examination confirmed the diagnosis of calciphylaxis. Management included intensification of dialysis, discontinuation of warfarin, correction of calcium-phosphate imbalance, wound care, and targeted antibiotics. Despite the multidisciplinary intervention, the patient experienced prolonged healing complicated by secondary infection. This case underscores the diagnostic and therapeutic challenges associated with calciphylaxis and highlights the need for early recognition and individualized treatment to improve patient outcomes.

## Introduction

Calciphylaxis is a rare condition with a complex pathophysiology characterized by calcification, fibrosis of the vascular intima, and blood clots causing cutaneous necrosis; the condition carries a poor prognosis, with mortality around 50% in one year [1,2]. Calciphylaxis was first described in the literature in 1899 by two British doctors at Guy’s Hospital, Dr Bryant and Dr White, who observed a case of a 6-month-old infant who was generally wasted and developed right toe gangrene and shortly after died in hospital [3]. Historically, calciphylaxis was mentioned in the literature under various names, including calcific uremic arteriolopathy, metastatic calcinosis cutis, and necrotizing or calcifying panniculitis, not until 1962 when Dr Selye introduced the name calciphylaxis [4]. Calciphylaxis is typically associated with renal failure and causes significant sepsis. Diagnosis is mainly made by combining clinical findings and histopathological features [5].

## Case presentation

A 76-year-old male patient presented with a nonhealing ulcer over the posterior aspect of his right calf, persisting for three months. His medical history was significant for atrial fibrillation (AF), managed with over 30 years of warfarin therapy, and end-stage renal disease (ESRD), for which he underwent hemodialysis three times weekly.

The ulcer developed following trauma, when the patient struck his calf against a broken chair. He experienced worsening right lower limb swelling and pain one week before the presentation. He initially sought treatment from his general practitioner (GP), who prescribed a two-week course of flucloxacillin for suspected cellulitis. However, the symptoms persisted and progressively worsened. At the time of presentation, the patient was ambulatory with minimal support. Although his mobility had significantly declined over the past three months, he had previously been independently mobile. Due to a clinical suspicion of deep vein thrombosis (DVT), the GP referred him to the Same Day Emergency Care (SDEC) unit for further evaluation, including a Doppler ultrasound of the right lower limb.

**Figure 1:**
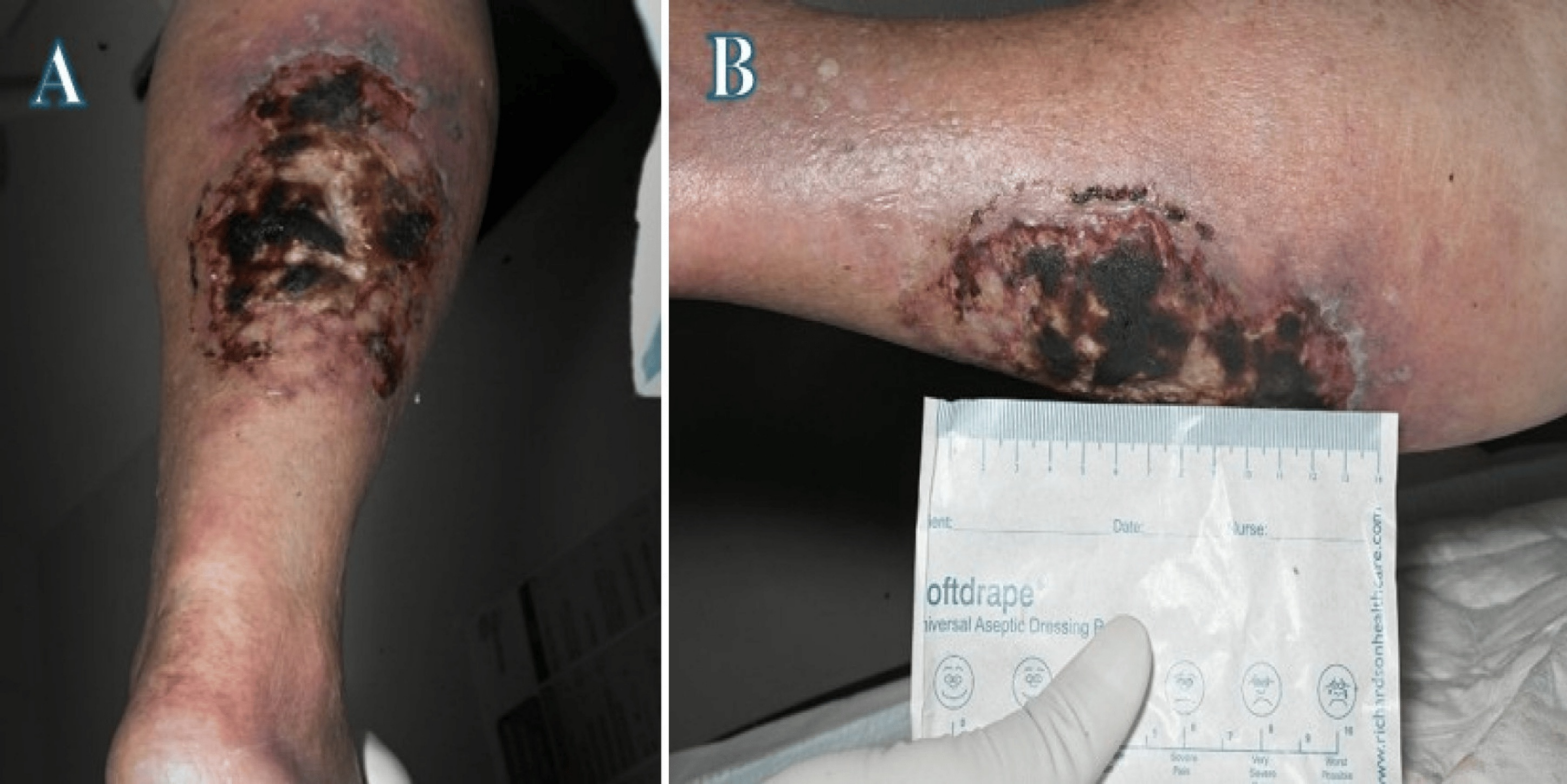
Right leg ulcer with calciphylaxis features of necrotic base and erythematous violaceous border (approximately 13 cm): (A) posterior view, (B) lateral view

On examination, the right calf was swollen, erythematous, and warm to the touch. The open ulcer measured approximately 13 cm long, was oval-shaped, and exhibited a necrotic base with violaceous borders (Figures 1A and 1B). Serous fluid was noted to be oozing from the wound, prompting a wound swab for microbiological assessment. There were no signs of neurological deficits or vascular compromise.

Laboratory investigations revealed chronic normocytic anemia, elevated C-reactive protein (CRP), hypercalcemia, hyperphosphatemia, and increased alkaline phosphatase (ALP). Procalcitonin was raised, while D-dimers (a fibrin degradation product) and white cell counts were within normal ranges (Table 1).

**Table 1:** Blood test results

Test	Result	Normal Reference Range
Neutrophils	5	2.0-7.5 x 10^9^/L
D-Dimer (a fibrin degradation product)	145	<500 ng/mL FEU
Estimated glomerular filtration rate (eGFR)	9	>90 mL/min/1.73 m²
Procalcitonin	0.64	<0.1 ng/mL
Corrected calcium	2.80	2.2-2.6 mmol/L
Phosphate	1.68	0.8-1.5 mmol/L
C-reactive protein (CRP)	81	<5 mg/L
Alkaline phosphate (ALP)	144	30-130 U/L
Sodium (Na)	136	135-145 mmol/L
Potassium (K)	4.4	3.5-5.0 mmol/L
Urea	13.7	2.5-7.8 mmol/L
Creatinine	498	60-110 µmol/L
Hemoglobin (Hb)	104	120-160 g/L (female), 130-180 g/L (male)
White cell count (WCC)	7	4.0-11.0 x 10^9^/L
Platelets	261	150-400 x 10^9^/L
Mean corpuscular volume (MCV)	96.8	80-100 fL

The clinical findings and laboratory results raised the possibility of calciphylaxis. Initial differential diagnoses also included warfarin-induced skin necrosis and cellulitis.

**Figure 2:**
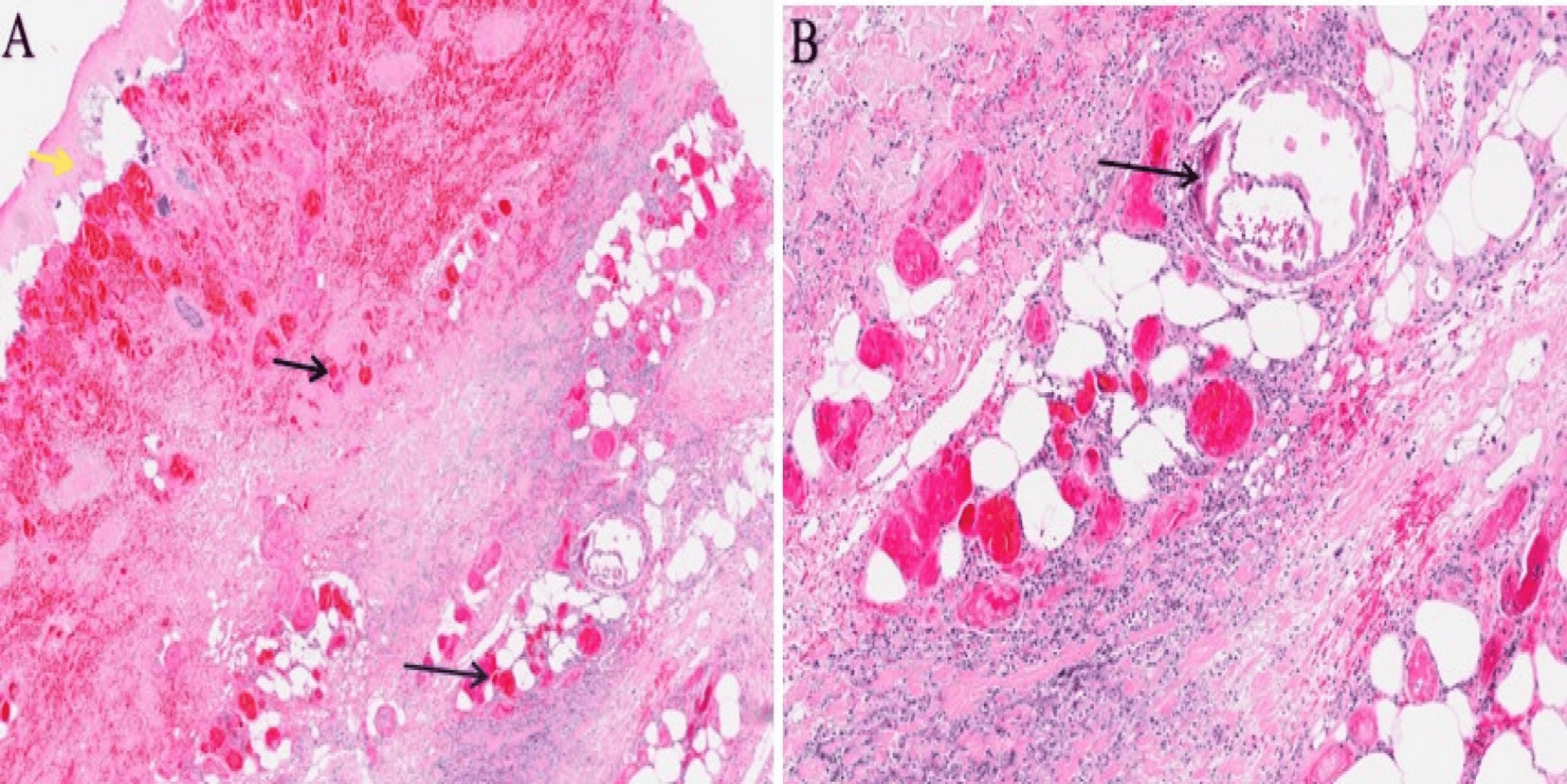
Histopathological image of the skin biopsy (A) Yellow arrow, epidermal necrosis; black arrows, hemorrhage within dermis and subcutaneous fat. Hematoxylin and eosin (H&E) stain: magnification ×10. (B) Black arrow, calciphylaxis involving the subcutaneous fat. Hematoxylin and eosin (H&E) stain: magnification ×20.

Multidisciplinary input was sought, and the hematology and vascular surgery teams recommended dermatological evaluation. Subsequently, the dermatology team performed a punch biopsy of the ulcer, which showed epidermal necrosis and calciphylaxis, confirming a diagnosis of calcific uremic arteriopathy (calciphylaxis) (Figure 2).

Management involved discontinuing warfarin and initiating alternative anticoagulation strategies. The wound swab showed heavy growth of colonizing flora without evidence of acute infection. The patient was advised to follow up with his hemodialysis team for ongoing chronic kidney disease management and dermatology team for ongoing care and consideration for sodium thiosulfate if deemed appropriate.

This case highlights the diagnostic challenges and the necessity of a multidisciplinary approach in identifying calciphylaxis, a rare but serious complication in patients with ESRD on long-term warfarin therapy. 

## Discussion

Calciphylaxis represents a rare, yet profoundly debilitating disorder characterized by cutaneous arteriolar and vascular calcification predominantly affecting small- and medium-sized vessels. Endothelial injury and the subsequent formation of microthrombi further diminish blood flow, resulting in luminal narrowing and occlusion. These pathophysiological alterations precipitate notable dermatologic manifestations, leading to tissue ischemia and infarction, manifesting as painful skin lesions [6]. Calciphylaxis is associated with considerable morbidity, attributed to severe pain, delayed wound healing, and frequent hospitalizations. Moreover, it carries a high mortality risk, with one-year mortality rates exceeding 50%, commonly stemming from complications such as sepsis [7]. Calciphylaxis lesions typically manifest as sudden and rapidly progressing painful eruptions, which may be solitary or multiple and predominantly affect the lower extremities [8].

The clinical presentation of calciphylaxis lesions encompasses early features of livedo reticularis and stellate or reticulate purpura, along with erythematous papules, plaques, or nodules. Advanced lesions often exhibit a rapidly progressing stellate purpuric pattern with central cutaneous necrosis. Alternatively, lesions may occasionally present as bullae. Ulceration, considered a late-stage manifestation, demonstrates impaired healing and is frequently complicated by secondary infection, contributing to an elevated mortality risk [9]. 

Calciphylaxis primarily affects patients with ESRD undergoing dialysis, with an incidence rate of 3.49 per 1,000 patient-years among patients receiving maintenance hemodialysis according to one study [10]. Although sporadic cases occur in those with acute renal failure, normal renal function, or earlier stages of chronic kidney disease (non-uremic calciphylaxis) [11], the pathogenesis of calciphylaxis remains poorly understood. Still, it is likely multifactorial, involving various comorbid factors such as chronic renal failure, obesity, diabetes mellitus, hypercalcemia, hyperphosphatemia, elevated calcium-phosphate product, secondary hyperparathyroidism, and hypercoagulable states. Despite the prevalence of these abnormalities in ESRD patients, calciphylaxis is relatively rare. Risk factors include calcium supplements, calcium-based phosphate binders, active vitamin D, warfarin, corticosteroids, iron therapy, and trauma associated with subcutaneous insulin or heparin injections [12]. 

There is evidence suggesting that the uremic milieu may contribute to calcification by inhibiting endogenous inhibitors of calcification, including alpha2-Heremans-Schmid glycoprotein/fetuin-A (AHSG), osteopontin, and matrix Gla protein, although these hypotheses remain speculative [13].

Warfarin, a vitamin K antagonist and well-established anticoagulant, impedes the carboxylation and activation of vitamin K-dependent clotting factors, potentially lowering protein C concentrations and inducing a procoagulant state in calcified vessels. Recent research indicates that endogenous inhibitors of vascular calcification, such as Matrix Gla Protein, also depend on vitamin K for their activation. Consequently, patients on warfarin therapy may experience reduced inhibition of vascular calcification due to decreased activation of these proteins [14].

The diagnostic approach for calciphylaxis primarily entails conducting a skin biopsy, which is complemented by laboratory analyses. These analyses include assessing renal function and electrolyte levels and measuring serum concentrations of calcium, phosphate, alkaline phosphatase, and albumin. Additionally, evaluating parathyroid function involves determining serum parathyroid hormone (PTH) levels. Furthermore, investigating coagulation factors, markers of inflammation (such as erythrocyte sedimentation rate (ESR) and CRP), and screening for underlying vasculitis is achieved through tests for antineutrophilic antibody and antineutrophil cytoplasmic antibody [15].

Histological examination in calciphylaxis commonly reveals calcification within the media and intima of the small- and medium-sized arterioles, frequently accompanied by notable intimal hyperplasia and fibrosis. Additionally, a mixed inflammatory infiltrate is commonly observed. Occasionally, subcutaneous calcium deposits associated with panniculitis and fat necrosis are present. Moreover, evidence of vascular microthrombi may be identified [16].

The management of calciphylaxis is multidisciplinary in nature, focusing on various aspects including primary prevention, appropriate wound care, pain management, and hormonal and mineral balance. It is essential to address exacerbating factors and eliminate triggering elements. This entails discontinuation of calcium supplementation, vitamin D supplementation, and warfarin therapy. In patients with an elevated calcium-phosphate product, efforts should be made to normalize serum calcium and phosphate concentrations promptly and safely. Augmenting the frequency or duration of dialysis sessions may yield some benefits [17].

Antibiotic therapy administered strategically may confer advantages in management. Additionally, hyperbaric oxygen therapy may prove beneficial in promoting wound healing in select cases [18,19].

The utilization of intravenous sodium thiosulfate therapy has garnered interest, supported by limited reports demonstrating its efficacy in managing calcific nephrolithiasis among nonuremic individuals and treating tumoral calcinosis in hemodialysis patients. Its mechanism involves enhancing the clearance of calcific deposits during hemodialysis sessions [20].

## Conclusions

Calciphylaxis remains a diagnostically and therapeutically challenging rare condition, marked by high morbidity and mortality, particularly among patients with ESRD. This case underscores the critical importance of early clinical recognition, histopathological confirmation, and prompt initiation of a multidisciplinary management strategy. Therapeutic measures, including correction of mineral imbalances, cessation of exacerbating agents such as warfarin, optimization of dialysis, and the use of intravenous sodium thiosulfate, may mitigate disease progression and improve wound healing. Nevertheless, outcomes remain poor, largely due to the high risk of secondary infection and sepsis. Given the rarity and complexity of calciphylaxis, further research is warranted to elucidate its pathogenesis, identify robust biomarkers, and develop targeted therapies. Heightened clinical vigilance and individualized patient care remain paramount in improving prognostic trajectories in this devastating disorder.
